# Prevalence of Dengue Serotypes and Its Correlation With the Laboratory Profile at a Tertiary Care Hospital in Northwestern India

**DOI:** 10.7759/cureus.15029

**Published:** 2021-05-14

**Authors:** Aviral Gupta, Puneet Rijhwani, Manish R Pahadia, Anchin Kalia, Shrikant Choudhary, Dharam P Bansal, Deepak Gupta, Pradeep Agarwal, Ram K Jat

**Affiliations:** 1 Department of General Medicine, Mahatma Gandhi Medical College and Hospital, Jaipur, IND

**Keywords:** dengue, serotype, dengue virus, denv, rt-pcr, thrombocytopenia

## Abstract

Background and aim

Dengue fever is an emerging arboviral public health problem in a large endemic population in the tropical and sub-tropical areas of the world, with varying degrees of clinical presentation. This study was aimed at analyzing the clinical and laboratory dynamics of the four dengue serotypes.

Methods

This institutional review board (IRB)-approved hospital-based observational study was performed with 100 in-patients with dengue infection above 12 years of age, without co-morbidities or known malignancy, in a tertiary care center in Northern India.

Results

Out of 100 patients, four had concurrent infection with two serotypes. Dengue virus serotype-2 (DENV 2) was the most common serotype (34%) and had the maximum percentage of cases of severe dengue (20.6%). The mean total leukocyte count did not differ between the serotypes. DENV 4 had a significantly higher mean neutrophil percentage and a significantly lower mean lymphocyte percentage than DENV 1 (p-value 0.001 and 0.02, respectively), with a higher percentage of cases of severe dengue (20% vs 14.3%, non-significant). Thrombocytopenia was present in all serotypes of infection. There was a significant difference in the derangement of liver function in DENV 2, 3, and 4 as compared to DENV 1. Mean serum albumin levels were significantly lower in DENV 3 and 4 infections. Cases with co-infection had a much higher derangement of liver function and lower mean serum albumin than infections with a single serotype. The mean blood urea and creatinine levels were in the normal range for all serotypes. No mortality occurred in our study.

Conclusion

DENV 2 is the most common serotype with maximum severity at our hospital. DENV 2 and DENV 4 have a high percentage of cases with severe dengue (20.6% and 20%, respectively). The mean lymphocyte percentage was significantly lower while hepatic involvement and hypoalbuminemia were greater in DENV 4. Initial serotyping in patients with dengue can help monitor the epidemiological trends and help estimate the clinical and laboratory trends of the different serotypes of dengue infection. Particular care should be taken in patients with co-infection.

## Introduction

Dengue is a mosquito-borne arboviral disease and a major global public health threat in the tropical and sub-tropical regions of the world, specifically in the urban and semi-urban areas [1]. The World Health Organization (WHO) estimates that approximately 50-100 million individuals are infected with dengue annually, and more than 2.5 billion individuals live at risk in more than 100 countries of dengue transmission. Rapid unplanned urbanization and migration of population from rural to urban areas, lack of vector control, climatic changes, and poor sanitation facilities have contributed to fertile breeding areas for the dengue vector, *Aedes aegypti* [2]. The typical presentation and classification of severity of dengue is depicted in Appendix 1.

Dengue is caused by four different serotypes of the dengue virus (called DENV 1, DENV 2, DENV 3, and DENV 4), and infection can occur by any one or more than one of the four serotypes. Infection with any one dengue serotype provides lifelong homotypic immunity to that particular serotype. It is well-documented that subsequent infections with different dengue virus serotypes increase the risk of developing severe dengue [3].

Dengue is typically diagnosed by non-structural protein-1 (NS1) antigen capture assays, detectable up to nine days after symptom onset in primary infection. But patients with secondary infection have NS-1 detectable for a much shorter period due to an anamnestic response. Serological diagnosis by immunoglobulin G or immunoglobulin M antibody capture assay suffers from cross-reactivity with other flavivirus infections. Molecular methods, such as reverse transcription-polymerase chain reaction (RT-PCR) provides a same-day diagnosis of DENV during the acute phase of the disease (phase with viremia) and can also detect the serotype (even in patients with secondary infection). PCR-based techniques are sensitive, specific, fast, less complicated, and cheaper than virus isolation methods [4].

Several studies have previously analyzed the clinical and epidemiological profile of dengue infection [3,5-9]. In this cross-sectional study, we aimed to study the different dengue serotypes and their clinical severity and biochemical laboratory profiles in patients with dengue, in an urban tertiary care center.

Aims and objectives

The aims and objectives of the current study are to (1) study the prevalence of dengue serotypes; (2) determine the rates of co-infection; and (3) correlate the serotypes with the degree of thrombocytopenia, total and differential leucocyte count, and derangement of hepatic and renal function.

## Materials and methods

The study was conducted in the Department of General Medicine of Mahatma Gandhi Medical College and Hospital, Jaipur. One hundred (100) patients admitted (in-patient) in the department with dengue fever were selected for the study from the period of January 2019 to June 2020. The study was approved by the Institutional Ethical Committee and informed consent was obtained from all the participants.

Patient selection

Patients over 12 years of age testing positive for dengue by reverse transcription-polymerase chain reaction (RT-PCR) were included in this cross-sectional study. All patients had a detailed medical history and a thorough physical examination at the time of admission.

Patients with co-morbidities, including diabetes mellitus, hypertension, hypothyroidism, liver disease, and chronic kidney disease while patients with a known malignancy, hematological disorder, or using any drug that may cause thrombocytopenia were excluded.

Laboratory evaluation required

Total leucocyte count (TLC), differential leucocyte count (DLC), platelet count, aspartate aminotransferase (AST), alanine aminotransferase (ALT), serum albumin, blood urea nitrogen (BUN), and serum creatinine were obtained at admission. The dengue serotype was confirmed by ribonucleic acid (RNA) amplification by ABI 7500 Fast Dx (Applied Biosystems, Foster City, California) RT-PCR. Patients were classified into non-severe dengue without warning signs/non-severe dengue with warning signs/severe dengue as per the WHO classification (Appendix 1).

Data analysis

For descriptive analyses, numbers and percentages were used to express categorical variables. Means with standard deviations were used to express continuous variables.

The Kruskal-Wallis test was used for the ordinal variables and the one-way analysis of variance (ANOVA) test for continuous variables. Post hoc analysis was performed using the Bonferroni method. A p-value of <0.05 was considered statistically significant. As the number of patients in the co-infection group was small (four patients), this group was excluded from the analysis of correlation with laboratory profile and described separately. All the statistics were performed using IBM Statistical Product and Service Solutions (SPSS) v 26 (IBM Corp. Armonk, NY).

## Results

Table 1 shows that 21 patients had dengue infection with serotype-1, 34 patients with serotype-2, 26 patients with serotype-3, 15 patients with serotype-4 while three patients had co-infection with serotypes-1 and 3, and one patient had co-infection with serotypes-2 and 3. There was no statistically significant difference in the distribution of severity between the different serotypes of infection (p-value 0.491).

**Table 1:** Distribution of patients on the basis of serotype and severity *NS without W – Non-severe dengue without warning signs **NS with W – Non-severe dengue with warning signs DENV 1 - Dengue virus serotype-1, DENV 2 - Dengue virus serotype-2, DENV 3 - Dengue virus serotype-3, DENV 4 - Dengue virus serotype-4

Serotype	NS without W* n (%)	NS with W** n (%)	Severe n (%)	Total n (%)
DENV 1	08 (38.1)	10 (47.6)	03 (14.3)	21 (100)
DENV 2	18 (52.9)	09 (26.5)	07 (20.6)	34 (100)
DENV 3	14 (53.8)	10 (38.5)	02 (7.7)	26 (100)
DENV 4	04 (26.7)	08 (53.3)	03 (20)	15 (100)
Co-infection (1+3)	02 (66.7)	01 (33.3)	--	03 (100)
Co-infection (2+3)	--	01 (100)	--	01 (100)
TOTAL	46	39	15	100

Table 2 shows no significant difference in the mean leucocyte count in the groups (p-value 0.575) and a statistically significant difference in the mean neutrophil and lymphocyte percentages in the groups (p-value <0.001 and 0.004, respectively). Post hoc analysis indicated a significantly higher neutrophil percentage in serotype-4 as compared to serotypes-1 and 2 (p-value 0.001 and 0.016, respectively; and a significantly lower lymphocyte percentage in serotype-1 as compared to serotypes-3 and 4 (p-value 0.043 and 0.02, respectively).

**Table 2:** Mean total leucocyte count and neutrophil and lymphocyte percentages based on serotypes TLC - Total leucocyte count, DENV 1 - Dengue serotype-1, DENV 2 - Dengue serotype-2, DENV 3 - Dengue serotype-3, DENV 4 - Dengue serotype-4

Serotype	TLC (10^3^/mm^3^)	Neutrophils (%)	Lymphocytes (%)
DENV 1	6.71 + 2.99	55.48+9.80	33.10+ 8.15
DENV 2	6.58 + 2.69	59.94+ 8.67	31.37+ 7.41
DENV 3	7.43 + 3.01	65.47+9.93	26.13 +8.48
DENV 4	6.27 + 2.73	69.75+ 14.15	24.30+ 11.68

Table 3 shows no significant difference in the mean platelet count among the groups (p-value 0.190).

**Table 3:** Mean platelet count in the groups based on serotypes DENV 1 - Dengue serotype-1, DENV 2 - Dengue serotype-2, DENV 3 - Dengue serotype-3, DENV 4 - Dengue serotype-4

Serotype	Platelet count (10^3^/mm^3^)
DENV 1	48.24+23.71
DENV 2	55.97+22.86
DENV 3	63.54+26.77
DENV 4	53.33+23.26

Table 4 shows a statistically significant difference in the mean ALT levels and mean AST and serum albumin levels (one-way ANOVA, p-value <0.001, <0.001 and <0.001, respectively). Post hoc analysis (Bonferroni method) indicated significantly higher mean ALT and AST levels in serotypes-2, 3, and 4 as compared to serotype-1 (p-value <0.001, 0.002, 0.006 and <0.001, <0.001, <0.001, respectively). Mean serum albumin levels were significantly lower in serotypes-3 and 4 as compared to serotype-1 and 2 (p-value 0.002, 0.002 and <0.001, <0.001, respectively).

**Table 4:** Mean serum ALT, AST, and albumin levels in the groups based on serotypes AST - Aspartate aminotransferase, ALT - Alanine aminotransferase, DENV 1 - Dengue serotype-1, DENV 2 - Dengue serotype-2, DENV 3 - Dengue serotype-3, DENV 4 - Dengue serotype-4

Serotype	ALT (U/L)	AST (U/L)	Albumin (gm/dL)
DENV 1	40.86+11.64	48.14+11.01	3.87+0.63
DENV 2	74.32+21.30	91.09+22.68	3.89+0.56
DENV 3	63.50+21.63	84.92+28.05	3.28+0.46
DENV 4	64.13 + 24.69	85.87 + 24.92	3.19 + 0.40

Table 5 shows the mean blood urea nitrogen (BUN) and mean serum creatinine levels within the normal range in all groups.

**Table 5:** Mean serum BUN and creatinine levels in the groups based on serotypes BUN - Blood urea nitrogen, DENV 1 - Dengue serotype-1, DENV 2 - Dengue serotype-2, DENV 3 - Dengue serotype-3, DENV 4 - Dengue serotype-4

Serotype	BUN (mg/dL)	Creatinine (mg/dL)
DENV 1	20.63+3.26	1.11+0.22
DENV 2	23.16+4.96	1.09+0.15
DENV 3	17.44+ 6.00	1.37+0.25
DENV 4	20.69 +5.79	1.16 + 0.31

Table 6 shows the comparison of mean laboratory values in patients without co-infection versus those with co-infection. Both groups with co-infection had a higher mean neutrophil percentage, platelet count, AST, and ALT than the no co-infection group and lower lymphocyte percentage and albumin than the no co-infection group. Co-infection with serotypes-1 and 3 had a higher BUN and creatinine than the no co-infection group.

**Table 6:** Mean laboratory values in patients without co-infection versus those with co-infection TLC - Total leucocyte count, ALT - Alanine aminotransferase, AST - Aspartate aminotransferase, BUN - Blood urea nitrogen

Laboratory	No co-infection	Co-infection (1+3)	Co-infection (2+3)
TLC (10^3^/mm^3^)	6.79 + 2.84	6.51 + 1.32	14.03
Neutrophils (%)	62 + 11.20	77.87 + 12.55	89.7
Lymphocytes (%)	29.22 + 9.14	15.23 + 10.43	7.7
Platelet count (10^3^/mm^3^)	55.92 + 24.45	75.0 + 37.72	68.0
ALT (U/L)	62.48 + 23.52	233.67 + 100.07	155.0
AST (U/L)	79.21 + 27.98	346.0 + 199.73	220.0
Albumin (gm/ dL)	3.61 + 0.61	3.13 + 0.49	2.70
BUN (mg/dL)	20.67 + 5.50	24.95 +10.72	20.2
Creatinine (mg/dL)	1.18 + 0.25	1.30 + 0.31	1.20

All the patients recovered from the disease with no mortality, and hospital stay varied from two days to 11 days (Mean 4.92 + 1.796 days).

## Discussion

Dengue is one of the notable arboviral infections for its dramatic epidemiological change over the past 20 years and is a major public health problem worldwide, especially in the tropical and subtropical areas.

In our study, 100 patients with RT-PCR-confirmed dengue were studied. Out of those 100 cases, 60% were infected with either dengue serotype-2 (DENV 2) or dengue serotype-3 (DENV 3) (Table 1). Maximum cases (34%) of dengue serotype-2, followed by 26% cases of DENV 3, 21% cases of DENV 1, and 15% cases of DENV 4 were found. This is in concordance with other studies from Delhi, Uttar Pradesh, and Mumbai [5-7]. In our study, four patients had concurrent infection with two serotypes of the dengue virus. three patients had co-infection with serotypes-1 and 3, and one patient had co-infection with serotypes-2 and 3. Previously, a study from Delhi reported the first concurrent infections by different dengue serotypes [5].

On serotype correlation with severity (Table 1), we found maximum cases of severe dengue in DENV 2 (20.6%) and DENV 4 (20%). However, this difference in distribution was not statistically significant (p-value = 0.491). Racherla et al. [8] in 2018, found DENV 2 as the predominant serotype in their study with higher severity of infection with DENV 3 and DENV 4.

Mean TLC (Table 2) was within the normal limits in all serotypes of infection (excluding co-infection). But the mean neutrophil and lymphocyte percentages (Table 2) were significantly different between the groups (p-value <0.001 and 0.004, respectively). These results are in concordance with a study conducted by Wardhani et al. [9] in 2017. A previous study conducted in Thailand suggested the absence of leucopenia as a predictor of severe dengue [10]. They found mean lymphocyte values were within the normal range, though it is expected to rise during many viral infections. Post hoc analysis in our study indicated that patients infected with serotype-4 had a significantly lower lymphocyte percentage than serotype-1 (p-value = 0.02) while serotype-4 was also one of the serotypes with the highest percentage of severe dengue (20%). Our results are in contrast to the findings of previous studies, where there was a significant increase in the number of atypical lymphocytes in dengue fever while normal lymphocytes were essentially unchanged [11]. It has been hypothesized that leucopenia in dengue is caused by the destruction or inhibition of myeloid progenitor cells, which can be found on bone marrow examination showing mild hypo-cellularity in the first seven days of fever, followed by normal cellularity in the convalescent phase [12]. Interestingly, the patient with co-infection with serotypes-2 and 3 in our study had a much higher TLC, higher neutrophil percentage, and a lower lymphocyte percentage compared to other cohorts.

In our study, thrombocytopenia was present in all serotypes of dengue infection, with the lowest value (13x10^3^/mm^3^) noted in DENV 1 and DENV 3 (Table 3). There was a statistically non-significant difference in the platelet counts between the different serotypes of infection (p-value = 0.190). Chaloemwong et al. [13] found almost all patients had thrombocytopenia, but most of them were a non-severe form of dengue infection so the bleeding diathesis in their study was low (5.8%). Azin et al. [14] found thrombocytopenia occurring from the onset of symptoms in hemorrhagic and severe forms of dengue, and remained stable throughout the progression of the disease. Mean platelet counts for co-infection groups were higher than the mean for other groups in our study.

The mean ALT and AST levels were higher in co-infection with dengue viruses as compared to infection with a single serotype (Table 6), and they were significantly higher in serotypes-2, 3, and 4 as compared to serotype-1 (Table 4), indicating the possibility of a greater hepatotropic nature of serotypes-2, 3, and 4. Mean AST was higher than mean ALT for all serotypes in our study. Ferede et al. [15] found elevation of AST and ALT in 45.1% and 17.6 % of the cases, respectively, with the relative elevation of AST in a greater proportion of cases than ALT. This is in agreement with another study by Mariappan T [16] with elevated AST and ALT levels in 68.5% and 39.2% of the cases, respectively. Similar findings were also reported (elevated AST in 72.7% and ALT in 27.3% of cases) in another study [17]. The hypothesized reason is that though the dengue virus is hepatotropic, it damages other organs as well; hence, the observed pattern could be explained due to the excess release of AST from damaged muscle cells (non-hepatic source) during infection, which leads to more elevation of AST than ALT. ALT is mainly associated with hepatocytes, with minor activity in cardiac and skeletal muscle. The concentration of AST is much higher in erythrocytes, kidney and brain tissue, and cardiac and skeletal muscle; it is usually raised because of damage to these sources and in response to hepatic damage [17].

Our study showed overall lower values of albumin in all serotypes of infection (Table 4), but in cases of co-infection, hypoproteinemia was more evident (Table 6). There was a significantly lower mean serum albumin level in serotypes-3 and 4 as compared to serotypes-1 and 2. It could be probable that the complex interaction between the virus, host immune response, and endothelial cells may affect the barrier integrity and functions of the vascular endothelial cells, leading to plasma leakage causing hypoproteinemia. Ferede et al. showed similar results [15]. According to their study, hypoproteinemia was observed in 21.6% of the cases.

## Conclusions

Our study shows that at our tertiary care hospital in Jaipur, Rajasthan, the most prevalent dengue serotypes were DENV 2 and DENV 3. Four cases of concurrent infection with two serotypes of dengue virus were also present. The maximum percentage of cases of severe dengue were present in DENV 2 and DENV 4 infection, though not statistically significant. There was no significant difference in TLC in the four serotypes. The mean lymphocyte percentage was lower in DENV 4 as compared to DENV 1, and DENV 4 also had a higher percentage of cases with severe dengue (20% vs 14.3%, non-significant). Thrombocytopenia was seen in all serotypes, with no significant difference in the degree of thrombocytopenia between the serotypes. Significantly higher mean AST and ALT levels were noted in DENV 2, 3, and 4, particularly in cases with co-infection. AST levels were higher than ALT levels for all serotypes, and significantly lower mean serum albumin levels were noted in DENV 3 and 4. Mean BUN and creatinine were within the normal range in all serotypes of infection. Testing the serotype of dengue infection at the time of diagnosis should be considered, as it can help the clinician be vigilant of patients infected with dengue serotypes with higher disease severity (DENV 2 and DENV4) or patients with co-infection. The possible greater hepatotropic nature of DENV 2, 3, and 4 needs to further evaluated with larger studies. The confirmation of the dengue virus and the infecting serotype by RT-PCR might be a potential line of approach in the evaluation and management of dengue. Interesting findings were seen in the co-infection patients, however, its impact on severity and course of illness cannot be assessed accurately due to the very small sample size.

## Figures and Tables

**Figure 1:**
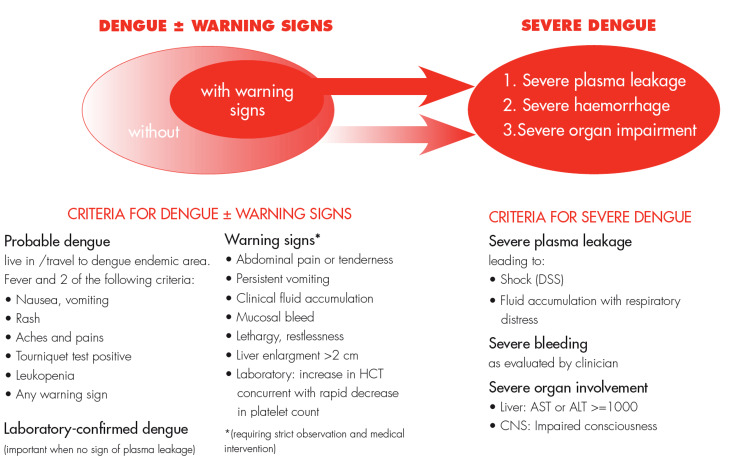
Appendix 1 - Dengue case classification and levels of severity Source: [18]
